# Antibacterial Activity of Salicylanilide 4-(Trifluoromethyl)benzoates

**DOI:** 10.3390/molecules18043674

**Published:** 2013-03-25

**Authors:** Martin Krátký, Jarmila Vinšová, Eva Novotná, Jana Mandíková, František Trejtnar, Jiřina Stolaříková

**Affiliations:** 1 Department of Inorganic and Organic Chemistry, Faculty of Pharmacy, Charles University, Heyrovského 1203, 500 05 Hradec Králové, Czech Republic; E-Mail: martin.kratky@faf.cuni.cz; 2 Department of Biochemical Sciences, Faculty of Pharmacy, Charles University, Heyrovského 1203, 500 05 Hradec Králové, Czech Republic; E-Mail: eva.novotna@faf.cuni.cz; 3 Department of Pharmacology and Toxicology, Faculty of Pharmacy, Charles University, Heyrovského 1203, 500 05 Hradec Králové, Czech Republic; E-Mails: jana.mandikova@faf.cuni.cz (J.M.); frantisek.trejtnar@faf.cuni.cz (F.T.); 4 Laboratory for Mycobacterial Diagnostics and Tuberculosis, Regional Institute of Public Health in Ostrava, Partyzánské náměstí 7, 702 00 Ostrava, Czech Republic; E-Mail: Jirina.Stolarikova@zu.cz

**Keywords:** antibacterial activity, antimycobacterial activity, cytotoxicity, isocitrate lyase inhibitor, multidrug-resistant tuberculosis, salicylanilide ester, 4-(trifluoromethyl)benzoic acid ester

## Abstract

The development of novel antimicrobial agents represents a timely research topic. Eighteen salicylanilide 4-(trifluoromethyl)benzoates were evaluated against *Mycobacterium tuberculosis*, *M. avium* and *M. kansasii*, eight bacterial strains including methicillin-resistant *Staphylococcus aureus* (MRSA) and for the inhibition of mycobacterial isocitrate lyase. Some compounds were further screened against drug-resistant *M. tuberculosis* and for their cytotoxicity. Minimum inhibitory concentrations (MICs) for all mycobacterial strains were within 0.5–32 μmol/L, with 4-chloro-2-[4-(trifluoromethyl)phenylcarbamoyl]phenyl 4-(trifluoromethyl)benzoate superiority. Gram-positive bacteria including MRSA were inhibited with MICs ≥ 0.49 μmol/L, while Gram-negative ones were much less susceptible. Salicylanilide 4-(trifluoromethyl)benzoates showed significant antibacterial properties, for many strains being comparable to standard drugs (isoniazid, benzylpenicillin) with no cross-resistance. All esters showed mild inhibition of mycobacterial isocitrate lyase and four compounds were comparable to 3-nitropropionic acid without a direct correlation between *in vitro* MICs and enzyme inhibition.

## 1. Introduction

The inappropriate use or misuse of established antimicrobial drugs has led to the increasing emergence of microbial resistance. The possible loss of the global control of infectious diseases represents a serious threat for public health, since resistance has been described for many important human pathogens.

Tuberculosis (TB) is responsible for millions of human deaths and about 1.4 million people died from TB in 2010 [1]. The drug-resistant TB forms, especially multidrug-resistant and extensively drug-resistant tuberculosis (MDR- and XDR-TB), bring grave problems. MDR-TB consists in a resistance to at least isoniazid (INH) and rifampicin (RIF), the most effective first-line oral agents, while XDR-TB is MDR-TB in combination with both resistance to any fluoroquinolone and at least one second-line injectable drug (kanamycin, amikacin, capreomycin) [2]. Latent TB and also coincidence of TB with HIV-infection signify other principal impetuses for novel antimycobacterial drugs.

Contemporary research trends are focused, *i.e.*, on the identification of unique mycobacterial pathways. Isocitrate lyase (ICL), an essential enzyme for the metabolism of fatty acids which splits isocitrate to succinate and glyoxylate, is one of two glyoxylate shunt enzymes. This pathway replenishes tricarboxylic acids cycle intermediates during growth on C_2_ substrates and is lacking in vertebrates. Disruption of *icl* gene attenuated bacterial persistence and virulence without affecting the bacterial growth during the acute phase of infection. ICL might contribute to mycobacterial adaptation to hypoxia. Conventional anti-tuberculosis drugs exhibit mostly an insufficient activity against slow- or non-growing (latent) mycobacteria. This is thought to be an important reason for the length of anti-tuberculosis therapy [3]. Novel ICL inhibitors may bring the shortening of TB treatment course and targeting of ICL represents one of the up-to-date research topics [4,5].

Nosocomial infections are a major challenge because of the high rates of morbidity and mortality. Worldwide, the state of resistance increased considerably in both Gram-positive and Gram-negative pathogens. Methicillin-resistant *Staphylococcus aureus* (MRSA), vancomycin-resistant *Enterococcus* spp., *Klebsiella pneumoniae*, *Escherichia coli*, *Pseudomonas* spp. or *Acinetobacter baumannii* producing extended spectrum β-lactamases (ESBL) represent such a most common and problematic drug-resistant bacteria, which have been rapidly spread throughout the world [6].

Consequently, novel drugs and strategies to control microbial spread are of critical importance, especially molecules with unique mechanism of action and no structural similarity. They can replace or combine with conventional antibiotics to combat problematic strains. The question if a new drug should be broad-spectral rather than have a narrow-spectrum of antimicrobial activity is controversial [7].

Salicylanilides (2-hydroxy-*N*-phenylbenzamides) and their derivatives have been reported to share a significant antibacterial activity, especially against atypical mycobacteria, *Mycobacterium tuberculosis* (*Mtb.*) including drug-resistant strains, Gram-positive cocci including MRSA with minimum inhibitory concentrations (MICs) in micromolar range [8,9,10,11,12,13,14]. Additionally, salicylanilides inhibit moderately mycobacterial isocitrate lyase and methionine aminopeptidase [4], but they affect multiple targets within the cells of pathogens [4,8,14,15].

Salicylanilide scaffold represents still an investigated group of compounds with many interesting pharmacological properties. Recently, they have been reported, e.g., as potential agents with a significant activity against *Onchocerca volvulus* [15], *Toxoplasma gondii* parasite [16] or with anticancer features [17,18,19,20].

Salicylanilides derived from 4-(trifluoromethyl)aniline have exhibited predominantly a high antimycobacterial activity; in general, the introduction of CF_3_ moiety into the salicylanilide scaffold enhanced antimicrobial properties [4,9,10,11,12,13]. Salicylanilide benzoates revealed a notable activity especially toward MDR-TB [13] as well as antibacterial one [11]. Aromatic esters of 4-(trifluoromethyl)benzoic acid have been reported as a mild antibacterial agents against *Pseudomonas fluorescens* and *Bacillus subtilis*, since inactive for *S. aureus* and *E. coli* [21], and some salicylanilide 4-(trifluoromethyl)benzoates exhibited antifungal activity, especially against moulds with MICs ≥ 0.49 μmol/L [22]. Additionally, an increased lipophilicity may ameliorate passing through cell walls and biomembranes, that is why we selected highly lipophilic salicylanilide 4-(trifluoromethyl)benzoates for the searching of a potential antibacterial and antimycobacterial agents with expected lower MIC.

## 2. Results and Discussion

Salicylanilide 4-(trifluoromethyl)benzoates **1** were evaluated for their *in vitro* antimycobacterial and antibacterial activity, cytotoxicity and inhibition of mycobacterial isocitrate lyase.

### 2.1. Chemistry

**Scheme 1:**
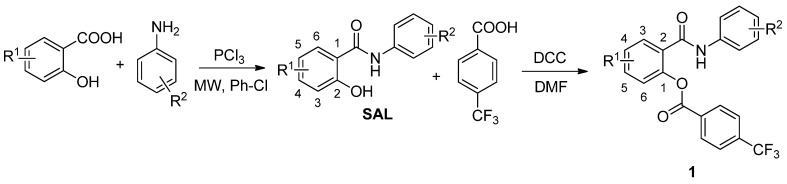
Synthesis of salicylanilides **SAL** and corresponding 4-(trifluoromethyl)benzoates **1** (R^1^ for esters: **1** = 4-Cl, 5-Cl, 4-Br; R^2^ = 3-Cl, 4-Cl, 3,4-diCl, 3-Br, 4-Br, 3-F, 4-F, 3-CF_3_, 4-CF_3_).

*Abbreviations*: MW: microwave irradiation (530 W, 600 rpm, 20 min); Ph-Cl: chlorobenzene; DCC: *N*,*N*'-dicyclohexylcarbodiimide; DMF: *N*,*N*-dimethylformamide.

The synthesis and characterization of the salicylanilide 4-(trifluoromethyl)benzoates (Scheme 1) were published recently [22]. Yield of esters synthesized via *N*,*N*'-dicyclohexylcarbodiimide coupling ranged from 49% up to 86%.

### 2.2. Antimycobacterial Evaluation

Table 1 summarizes the antimycobacterial activity of esters **1**, isoniazid and *para*-aminosalicylic acid, two comparative drugs. All of the 4-(trifluoromethyl)benzoates displayed a significant antimycobacterial activity, with MICs of 0.5–32 μmol/L against all strains, with **1o** and **1r** showing superiority (1–4 μmol/L). *Mycobacterium avium* showed the highest MIC values, up to 32 μmol/L. All esters displayed a better *in vitro* activity against four mycobacterial strains than PAS, a second-line oral drug sharing a structural similarity. Additionally, salicylanilide derivatives exhibited lower MICs than INH for *M. kansasii* 235/80 and *M. avium*; MIC values against *M. kansasii* 6509/96 were comparable or a slightly better in the case of esters. Only three esters (**1e**, **1o** and **1r**) showed equal activity like INH against *M. tuberculosis* (1 μmol/L). 4-(Trifluoromethyl)benzoic acid exhibited only a very mild intrinsic activity towards *M. kansasii* (MICs ≥ 250 μmol/L), whereas other mycobacteria were completely insusceptible at 1,000 µmol/L.

**Table 1:** Antimycobacterial activity of salicylanilide 4-(trifluoromethyl)benzoates **1**.

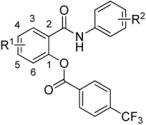																	
				MIC [μmol/L]													
	R^1^	R^2^		*Mtb.* 331/88		*M. avium* 330/88			*M. kansasii* 235/80					*M. kansasii* 6509/96				
				14 d	21 d	14 d		21 d	7 d		14 d	21 d		7 d		14 d	21 d
**1a**	4-Cl	3-Cl		**2**	4	8		16	**0.5**		2	4		**2**		8	8
**1b**	5-Cl	3-Cl		4	4	8		8	**1**		4	4		**2**		8	8
**1c**	4-Cl	4-Cl		**2**	**2**	4		8	**1**		2	2		**1**		4	4
**1d**	5-Cl	4-Cl		4	4	4		8	**1**		4	4		**1**		4	4
**1e**	4-Cl	3,4-diCl		**1**	**1**	4		4	**1**		2	4		**1**		**2**	4
**1f**	5-Cl	3,4-diCl		**1**	**2**	**2**		8	**1**		2	4		**1**		**2**	4
**1g**	4-Cl	3-Br		**1**	**2**	**2**		8	2		4	4		**1**		4	4
**1h**	5-Cl	3-Br		4	8	8		16	4		8	8		**2**		8	8
**1i**	4-Cl	4-Br		**2**	**2**	8		8	**1**		4	8		**2**		8	8
**1j**	5-Cl	4-Br		**2**	4	8		8	**1**		4	4		**2**		4	4
**1k**	4-Cl	3-F		8	8	16		32	8		16	16		8		16	16
**1l**	5-Cl	3-F		4	4	8		16	4		8	8		8		8	16
**1m**	4-Cl	4-F		8	8	16		32	4		16	16		8		16	16
**1n**	5-Cl	4-F		4	8	4		4	2		8	8		4		8	8
**1o**	4-Cl	4-CF_3_		**1**	**1**	**1**		4	**1**		2	2		**1**		**2**	**2**
**1p**	5-Cl	4-CF_3_		**2**	**2**	**2**		8	2		2	4		**1**		4	4
**1q**	4-Cl	3-CF_3_		**2**	**2**	4		16	2		4	8		**1**		4	8
**1r**	4-Br	4-CF_3_		**1**	**1**	**2**		4	**1**		2	4		**1**		**2**	4
**INH**				0.5–1	0.5–1	≥250	≥250		>250		>250	>250		2–4		4–8	8–16
**PAS**				62.5	62.5	32		125	125		1000	>1000		32		125	500
**CF_3_-BA**				>1,000	>1,000	>1,000		>1,000	1,000		>1,000	>1,000		250		1,000	1,000

INH = isoniazid; PAS = *p*-aminosalicylic acid; CF_3_-BA = 4-(trifluoromethyl)benzoic acid; Two best MICs for each strain are given in bold.

In general, the most active derivatives share a trifluoromethyl moiety (compounds **1o**–**r**) or two chlorines (compounds **1e**, **1f**) on the aniline ring; on the other side, fluorine atom provided only a minimal benefit. Esters of 5-chlorosalicylanilides showed a higher antimycobacterial activity than those derived from 4-chlorosalicylanilides, with fluorinated molecules **1k**–**n** being an exception.

Importantly, salicylanilide 4-(trifluoromethyl)benzoates **1** were assayed being predominantly more active than parent salicylanilides [23] – e.g., up to eight times for **1f**, **1n** and **1o** against *M. avium* or **1e** for *M. tuberculosis*. In some cases, esters and parent phenolic molecules share the same MIC values, e.g., **1b**, **1k** or **1d** for *M. tuberculosis*. Sporadically, the esterification led to the decreased potency; namely **1i**, **1k**, **1m** and **1o** against *M. kansasii*, **1m** and **1r** for *M. avium*. 5-Bromo-2-hydroxy-*N*-[4-(trifluoromethyl)phenyl]benzamide (**SAL-7**) represents only one salicylanilide, which esterification did not improve activity against any mycobacterial strain. Additionally, the most active compounds (*i.e.*, with at least one MIC against any strain ≤ 1 μmol/L) were evaluated against five MDR-TB strains and one XDR-TB strain with different resistance patterns (Table 2).

**Table 2:** MICs of salicylanilide 4-(trifluoromethyl)benzoates **1** towards MDR- and XDR-TB.

MIC [μmol/L]														
	R^1^	R^2^	*Mtb.* 7357/1988		*Mtb.* 9449/2006		*Mtb.* 53/2009		*Mtb.* 234/2005		*Mtb.* Praha 1		*Mtb.* Praha 131 (XDR-TB)	
			14 d	21 d	14 d	21 d	14 d	21 d	14 d	21 d	14 d	21 d	14 d	21 d
**1a**	4-Cl	3-Cl	4	4	4	4	4	4	4	4	4	4	4	4
**1b**	5-Cl	3-Cl	8	8	4	4	8	8	8	8	4	4	8	8
**1c**	4-Cl	4-Cl	2	2	2	2	4	4	4	4	2	2	2	2
**1d**	5-Cl	4-Cl	4	4	4	4	4	4	4	4	2	4	2	4
**1e**	4-Cl	3,4-diCl	**1**	2	2	2	2	4	**1**	2	**1**	2	2	2
**1f**	5-Cl	3,4-diCl	2	2	2	2	2	2	**1**	2	**1**	2	**1**	2
**1g**	4-Cl	3-Br	2	2	**1**	2	2	2	**1**	2	**1**	2	**1**	2
**1i**	4-Cl	4-Br	2	4	2	4	4	4	2	2	2	2	2	2
**1j**	5-Cl	4-Br	2	2	2	2	2	2	2	2	2	2	**1**	2
**1o**	4-Cl	4-CF_3_	**0.5**	**1**	**1**	**1**	**1**	**1**	**0.5**	**1**	**0.5**	**0.5**	**0.5**	**1**
**1r**	4-Br	4-CF_3_	**1**	**1**	**1**	**1**	**1**	**2**	**0.5**	**1**	**0.5**	**1**	**0.5**	**1**

MIC values of 1 μmol/L and lower are given in bold.

Importantly, all evaluated esters showed a significant activity towards MDR- and XDR-TB strains independently of the particular resistances. Interestingly, not only esters with the best activity towards *M. tuberculosis* (MICs ≤ 1 μmol/L) were assayed, as in previous studies [9,13,24], as even additional derivatives with MICs in the range of 2–4 μmol/L, were also powerful. All of these derivatives affected the growth of drug-resistant *M. tuberculosis* at equal or almost equal concentrations required for the inhibition of drug-sensitive one; drug-resistant strains were more susceptible to esters **1j**, **1o** and **1r** than *M. tuberculosis* H_37_Rv. These findings indicate that salicylanilide 4-(trifluoromethyl)benzoates do not share any cross-resistance with conventionally used drugs (INH, rifamycines, EMB, STM, OFX, clofazimine, aminoglycosides) and they may be prospective and perspective agents for combating drug-resistant TB.

4-(Trifluoromethyl)benzoates **1** possess potent *in vitro* antimycobacterial properties; although sharing a high lipophilicity, their efficacy did not surpassed esters with *N*-acetyl-L-phenylalanine [9] with MDR- and XDR-TB being an exception, but esters **1** exhibited lower MICs than, e.g., salicylanilide benzenesulfonates [10] or esters with *N*-benzyloxycarbonyl amino acids [25]. Carbamates showed better *in vitro* activity against *M. tuberculosis* including drug-resistant strains, while atypical mycobacteria were inhibited at similar concentrations [24]. When compared to closely related salicylanilide benzoates [13], the introduction of trifluoromethyl moiety into a benzoic acid ring did not improved activity, although the presence of this substituent in parent salicylanilides resulted unambiguously in the excellent antimycobacterial properties.

### 2.3. Isocitrate Lyase Inhibition Assay

Salicylanilide 4-(trifluoromethyl)benzoates were evaluated for their inhibition of mycobacterial ICL to elucidate their impact on one of the known targets for latent TB infection (Table 3). Compounds with dual effect on both the actively growing and latent mycobacterial populations may bring an appreciable benefit.

**Table 3:** ICL inhibition activity of salicylanilide 4-(trifluoromethyl)benzoates **1**.

	R^1^	R^2^	% ICL inhibition at 10 μmol/L (± standard deviation)
**1a**	4-Cl	3-Cl	18 ± 1.9
**1b**	5-Cl	3-Cl	13 ± 1.7
**1c**	4-Cl	4-Cl	12 ± 2.7
**1d**	5-Cl	4-Cl	12 ± 2.3
**1e**	4-Cl	3,4-diCl	18 ± 1.5
**1f**	5-Cl	3,4-diCl	17 ± 1.6
**1g**	4-Cl	3-Br	**27 ± 0.2**
**1h**	5-Cl	3-Br	21 ± 1.8
**1i**	4-Cl	4-Br	18 ± 1.2
**1j**	5-Cl	4-Br	14 ± 4.0
**1k**	4-Cl	3-F	**25 ± 0.8**
**1l**	5-Cl	3-F	20 ± 0.5
**1m**	4-Cl	4-F	**23 ± 1.4**
**1n**	5-Cl	4-F	17 ± 1.3
**1o**	4-Cl	4-CF_3_	13 ± 1.2
**1p**	5-Cl	4-CF_3_	17 ± 2.3
**1q**	4-Cl	3-CF_3_	19 ± 0.5
**1r**	4-Br	4-CF_3_	**23 ± 0.1**
3-NP			25 ± 4.1
INH			0

INH = isoniazid; 3-NP = 3-nitropropionic acid; Inhibition rate values comparable to 3-NP or higher are given in bold.

Salicylanilide 4-(trifluoromethyl)benzoates **1** showed 12%–27% inhibition of isocitrate lyase at 10 µmol/L concentration. Six esters demonstrated ≥ 20% enzyme inhibition rate (**1g**, **1h**, **1k**, **1l**, **1m**, **1r**) with superiority of the 3-bromoaniline derivative **1g**; this molecule also possessed higher potency than 3-NP, a well-known standard. Derivatives of 5-chlorosalicylic acid mostly exhibited higher potency than those related to 4-chlorosalicylic acid (**1a** *vs.* **1b**, **1g** *vs.* **1h**, **1k** *vs.* **1l**, *etc.*). The substitution of aniline ring at the position 3 is linked with enhanced enzyme inhibition when compared to 4-substituted anilines (e.g., **1a** *vs.* **1c**, **1g** *vs.* **1i**, **1o** *vs.* **1q**).

Salicylanilide 4-(trifluoromethyl)benzoates **1** produced significantly and consistently higher ICL-1 inhibition than previously reported salicylanilide derivatives – salicylanilides, their pyrazinoates, benzoates, benzenesulfonates or esters with *N*-acetyl-L-phenylalanine [4].

The additional ICL inhibition assay was performed with the derivatives at the concentration of 100 µmol/L; nevertheless, some of the compounds precipitated in the medium after a very short period during the investigation. This effect at the higher concentration may be render due to a high lipophilicity.

Obviously, even though salicylanilide 4-(trifluoromethyl)benzoates **1** exhibited a significant ICL-1 inhibition, their concentration values are higher (IC_50_ > 10 µmol/L) in comparison with MICs necessary for the mycobacterial growth arrest. Importantly, the inhibition of isocitrate lyase does not facilitate the elimination of mycobacteria during the acute phase of infection – in contrast to the chronic phase [3]. Therefore there is not a direct relationship of *in vitro* MICs of salicylanilides towards growing mycobacteria and the ICL inhibition, especially when the suppression of ICL may affect the slowly- or non-growing mycobacterial subpopulations existing on the nutrition poor substrates. Thus, the salicylanilide derivatives share a complex and still not fully elucidated mechanism of action for actively growing mycobacteria, since more cellular targets have been identified for this chemical group (e.g., [4,5,15]). Further experiments dealing with the mechanism(s) of action are appropriate.

### 2.4. Antibacterial Evaluation

Table 4 overviews the antibacterial activity. For the remaining ten esters (**1a**, **1c**, **1d**, **1f**, **1g**, **1i**, **1l**, **1n**, **1q**, **1r**), whose MICs are not reported, it was not possible to determine them. Similarly as in previously reported antifungal assay [22], they could not be dissolved in the testing medium well or they precipitated rapidly in them due to an escalated lipophilicity. 4-(Trifluoromethyl)benzoic acid showed no antibacterial action under our conditions (MIC > 500 µmol/L).

All soluble derivatives showed a very good activity against Gram-positive cocci, in some cases (**1e**, **1j**, **1p**, particularly **1b**, **1h** and **1k**) even excellent MICs lower than 1 μmol/L, despite the methicillin-resistance. Based on MIC values, these esters act likely mainly as bactericidal agents. MICs against *S. aureus* were comparable to benzylpenicillin and, moreover, 4-(trifluoromethyl)benzoates **1** exceeded its activity for MRSA, *S. epidermidis* and partly for *Enterococcus* (**1e**, **1j**, **1p**). Among Gram-negative bacteria, only *E. coli* possessed a partial susceptibility from 31.25 μmol/L (**1p**), while the growth of *P. aeruginosa* and both strains of *K. pneumoniae* was not reduced up to the concentration of 500 μmol/L.

Due to a limited number of assayed esters, it is difficult to discuss the structure-activity relationship. In the pair of **1o** and **1p**, the 5-chloro derivative (**1p**) showed lower MICs and when compared **1k** *vs.* **1m**, 3'-F moiety resulted in a higher activity.

Based on limited data, 4-(trifluoromethyl)benzoates displayed more significant activity against Gram-positive bacteria than salicylanilide benzoates [11], pyrazinoates [12] or esters with *N*-acetyl-L-phenylalanine [9].

**Table 4:** MICs of salicylanilide 4-(trifluoromethyl)benzoates **1** towards selected bacteria.

MIC/IC_90_ [μmol/L]												
	R^1^	R^2^	*S. aureus* CCM 4516/08		MRSA H 5996/08		*S. epidermidis* H 6966/08		*Enterococcus* sp. J 14365/08		*E. coli* CCM 4517	
			24 h	48 h	24 h	48 h	24 h	48 h	24 h	48 h	24 h	48 h
**1b**	5-Cl	3-Cl	**0.98**	1.95	**0.98**	1.95	**0.98**	1.95	7.81	15.62	>250	>250
**1e**	4-Cl	3,4-diCl	**0.49**	**0.98**	**0.49**	**0.98**	**0.49**	**0.98**	**0.49**	3.9	>500	>500
**1h**	5-Cl	3-Br	**0.98**	**0.98**	**0.49**	**0.98**	**0.49**	**0.98**	31.25	250	>500	>500
**1j**	5-Cl	4-Br	**0.49**	**0.98**	**0.49**	**0.98**	**0.49**	**0.98**	**1.95**	7.81	250	>250
**1k**	4-Cl	3-F	3.9	3.9	**0.98**	1.95	3.9	3.9	31.25	>250	>250	>250
**1m**	4-Cl	4-F	7.81	7.81	15.62	15.62	31.25	>500	500	>500	>500	>500
**1o**	4-Cl	4-CF_3_	3.9	7.81	3.9	7.81	3.9	7.81	7.81	62.5	>125	>125
**1p**	5-Cl	4-CF_3_	**0.49**	**0.98**	**0.49**	**0.98**	**0.49**	**0.98**	**0.49**	3.9	**31.25**	250
**PNC**			0.98	0.98	62.5	125	250	250	7.81	15.62	>500	>500

MRSA: methicillin-resistant *Staphylococcus aureus*; PNC: benzylpenicillin; One or two best MIC(s) for each strain are given in bold.

### 2.5. Cytotoxicity Evaluation

Since salicylanilide derivatives have displayed cytotoxicity for human cells (e.g., [4,9,20,24]), we examined *in vitro* cytotoxicity of six 4-(trifluoromethyl)benzoates, which shared the highest rate of MDR-TB growth suppression (**1e**, **1f**, **1g**, **1j**, **1o**, **1r**), as well as esters with the lowest MICs for *Staphylococcus* sp. (**1e**, **1j**, **1p**). For comparison, seven parent salicylanilides (**SAL-1** – **SAL-7**) and 4-(trifluoromethyl)benzoic acid were also investigated in the liver HepG2 cells model (Table 5). The parameter IC_50_ was used as a measure of toxicity, which allowed the quantitative comparison of the toxicity among the tested compounds. 

IC_50_ of 4-(trifluoromethyl)benzoates **1** were within the range 1.33–4.82 μmol/L and IC_50_ of parent salicylanilides of 0.36–1.98 μmol/L. For all pairs salicylanilide and its 4-(trifluoromethyl)benzoate, the esterification alleviated mildly the strong cytotoxicity of the parent salicylanilides containing free phenolic group, which is probably mainly responsible for the cytotoxicity. Its temporary masking leads to the derivatives with somewhat higher IC_50_ values – from 1.2-fold (**1r**) up to 3.7-fold for **1e** and **1p**, but still in similar concentration range. 4-(Trifluoromethyl)benzoic acid was many time less toxic with IC_50_ of 563.7 µmol/L.

Selectivity indexes (SI) ranges from 0.1 up to 1.67 for *M. tuberculosis* for parent salicylanilides, while esters with a significant activity against MDR-TB showed SI of 0.89–4.46 for drug-sensitive *M. tuberculosis* and of 0.78–4.46 for drug-resistant strains. SI values for *S. aureus* were within 0.17–9.84; SI of **1j** almost reached the value of 10, which is generally considered being borderline. With respect to the purposed cytotoxicity reduction of the parent salicylanilides, the results are not satisfactory.

**Table 5:** IC_50_ and SI for selected salicylanilide 4-(trifluoromethyl)benzoates **1**.

	R^1^	R^2^	IC_50_ [µmol/L] HepG2	SI for *Mtb.* H_37_Rv 331/88		SI for MDR-TB strains		SI for XDR-TB strain		SI for *Staphylococcus* sp.	
			24 h	14 d	21 d	14 d	21 d	14 d	14 d	24 h	48 h
**1e**	4-Cl	3,4-diCl	3.13	3.13	3.13	1.57–3.13	0.78–1.57	1.57	1.57	6.39	3.19
**1f**	5-Cl	3,4-diCl	4.46	4.46	2.23	2.23–4.46	2.23	4.46	2.23	-	-
**1g**	4-Cl	3-Br	2.63	2.63	1.32	1.32–2.63	1.32	2.63	1.32	-	-
**1j**	5-Cl	4-Br	4.82	2.41	1.21	2.24	2.24	4.82	2.41	9.84	4.92
**1o**	4-Cl	4-CF_3_	1.33	1.33	1.33	1.33–2.66	1.33–2.66	2.66	1.33	0.34	0.17
**1p**	5-Cl	4-CF_3_	1.77	0.89	0.89	-	-	-	-	3.61	1.81
**1r**	4-Br	4-CF_3_	2.03	2.03	2.03	2.03–4.06	1.02–2.03	4.06	2.03	-	-
**SAL-1**	5-Cl	3,4-diCl	0.84	0.21	0.10	-	-	-	-	-	-
**SAL-2**	4-Cl	3,4-diCl	1.80	0.45	0.45	-	-	-	-	-	-
**SAL-3**	5-Cl	3-Br	1.54	NT	NT	-	-	-	-	-	-
**SAL-4**	4-Cl	4-Br	1.98	0.5	0.5	-	-	-	-	-	-
**SAL-5**	5-Cl	4-CF_3_	0.36	0.18	0.18	-	-	-	-	-	-
**SAL-6**	4-Cl	4-CF_3_	0.69	0.17	0.17	-	-	-	-	-	-
**SAL-7**	5-Br	4-CF_3_	1.67	1.67	1.67	-	-	-	-	-	-
**CF_3_-BA**			563.70	-	-	-	-	-	-	-	-

CF_3_-BA = 4-(trifluoromethyl)benzoic acid. SI = IC_50_/MIC_100_; MIC values of salicylanilides against *M. tuberculosis* were taken from reference [23].

When compared to other salicylanilide esters, 4-(trifluoromethyl)benzoates showed about ten times higher cytotoxicity than salicylanilide carbamates [24], esters with *N*-acetyl-L-phenylalanine [9] or even more than esters with *N*-benzyloxycarbonyl amino acids [25], while it was approximately comparable with salicylanilide benzenesulfonates and benzoates [4].

However, salicylanilide esterification may represent one successful way to reduce their cytotoxicity for human cells as it was demonstrated especially for esters with *N*-protected α-amino acids [9,25] and carbamates [24]; it is necessary to search new type(s) of acid(s), because 4-(trifluoromethyl)benzoic acid brought a very mild and still insufficient benefit.

From other point of view, salicylanilides and their 4-(trifluoromethyl)benzoates may be considered as potential anticancer agents. One study [20] reported some salicylanilides being proposed and synthesized as potential cytotoxic agents against hepatocellular carcinoma. All of our salicylanilide derivatives showed IC_50_ values similar to the most active salicylanilides reported by Zhu *et al.* [20] (1.3 and 1.7 μmol/L); even three salicylanilides (**SAL-1**, **SAL-5**, **SAL-6**) were more active with 5-chloro-2-hydroxy-*N*-[4-(trifluoromethyl)phenyl]benzamide (**SAL-5**) being superior with IC_50_ of 0.36 μmol/L. Especially salicylanilide 4-(trifluoromethyl)benzoates **1** could be more convenient because previously it was demonstrated that some 3- and 4-(trifluoromethyl)benzoate esters of scaffolds with an intrinsic activity showed superiority among other esters [26,27].

The mechanism of cytotoxicity seems to be multiple – besides non-specific damage of cellular structures and function by the *in vivo* released phenolic group [8], it has been described a mild inhibition of human methionine aminopeptidase [4], inhibition of EGFR protein tyrosine kinases [17,19,20,28,29] or other human enzymes [18,19].

## 3. Experimental

### 3.1. Chemistry

The synthesis and characterization (m.p., IR and NMR spectra, elemental analyses) of the presented salicylanilide 4-(trifluoromethyl)benzoates [2-(phenylcarbamoyl)phenyl 4-(trifluoromethyl)benzoates; Scheme 1] **1a**–**r** was published recently by Krátký *et al.* [22].

### 3.2. Antimycobacterial Evaluation

All compounds were tested for their *in vitro* against *Mycobacterium tuberculosis* 331/88 (H_37_Rv; dilution of the strain was 10^−3^), nontuberculous mycobacteria: *Mycobacterium avium* 330/88 [resistant to INH, RIF, ofloxacin (OFX), and ethambutol (EMB); dilution 10^−5^], one strain of *Mycobacterium kansasii* 235/80 from the Czech National Collection of Type Cultures (CNCTC) (dilution 10^−4^) and a clinically isolated strain of *M. kansasii* 6509/96 (dilution 10^−5^). The micromethod for the determination of MICs was used. Antimycobacterial activities were determined in the Šula´s semisynthetic medium (SEVAC, Prague, Czech Republic). The tested compounds were added to the medium as solutions in dimethyl sulfoxide (DMSO). The following concentrations were used: 1000, 500, 250, 125, 62.5, 32, 16, 8, 4, 2, 1, 0.5, 0.25, and 0.125 μmol/L. The MICs were determined after incubation at 37 °C for 14 and 21 days, for *M. kansasii* additionally for 7 days. MIC (in μmol/L) was the lowest concentration at which the complete inhibition of mycobacterial growth occurred. The first-line anti-tuberculosis drug isoniazid (INH) and structurally similar *para*-aminosalicylic acid (PAS), a second-line oral agent, were chosen as the reference drugs.

The most active esters were evaluated at similar conditions and concentrations against six MDR-TB strains (dilution 10^−3^) with different resistance patterns. All strains are resistant to INH, RIF, rifabutine, and streptomycin (STM); an additional resistance was present in some cases: 7357/1998 and 234/2005 both resistant to INH, rifamycines, STM, EMB, and OFX; 9449/2006 resistant to INH, rifamycines, and STM; 53/2009 resistant to INH, rifamycines, STM, and EMB; Praha 1 resistant to INH, rifamycines, STM, EMB, and clofazimine; and Praha 131 resistant to INH, rifamycines, STM, EMB, OFX, gentamicin and amikacin (*i.e.*, XDR-TB strain). The following concentrations were used: 1000, 500, 250, 125, 62.5, 32, 16, 8, 4, 2, 1, 0.5, 0.25, and 0.125 μmol/L.

### 3.3. Isocitrate Lyase Inhibition Assay (ICL1)

The *Mycobacterium tubeculosis* H_37_Rv genomic DNA was used as a template. The *icl* gene (Rv0467) of 1.28 kb was amplified using PCR. The amplified DNA was cloned into the pET-28b(+) plasmid vector Novagen (Merck KGaA, Darmstadt, Germany) using *Nde*I and *Hind*III restriction sites. The recombinant plasmid was transferred into *E. coli* HB101. DNA sequencing was employed to confirm that the inserted coding sequence had no mutations. For bacterial expression, 25 mL culture volumes were inoculated with BL21(DE3) cells containing the recombinant plasmid and allowed to grow until an optical density of OD_595_ = 0.6 was achieved. Then, the culture was induced with 1 mmol/L isopropyl-*β*-D-thiogalactopyranoside solution and incubated at 30 °C for additional 4 h. The cells were harvested at 4 °C by centrifugation at 6,000 *g* for 10 min. The resulting pellet was resuspended in BugBuster Protein Extraction Reagent Novagen (Merck KGaA, Darmstadt, Germany). The cell debris was removed by centrifugation, and the histidine-tagged protein was purified using an Äkta purifier (Amersham Biosciences, Valley Stream, NY, USA). The purity of the protein was confirmed by SDS-polyacrylamide gel electrophoresis (SDS-PAGE) followed by Coomassie blue staining of the gel. The protein concentration was determined by the Bradford method [30].

The isocitrate lyase activity was assayed according to the protocol reported by Dixon and Kornberg [31]. The enzyme assay was optimised in the final volume of 100 μL using 96-well plates (NUNC, Schoeller, Prague, Czech Republic) and the final concentration of tested compound in the reaction mixture 10 μmol/L. The reaction buffer contained 50 mmol/L of KH_2_PO_4_, 4 mmol/L of MgCl_2_·6H_2_O, 4 mmol/L of phenylhydrazine hydrochloride, 12 mmol/L of L-cysteine, H_2_O and KOH to pH 7.0. The isocitrate cleavage was measured by the change in the absorbance at 324 nm, which is associated with the formation of glyoxylate phenyl hydrazone. Each tested compound was dissolved in DMSO to prepare a 1 mmol/L solution, and 1 μL of this solution was added to 93.9 μL of reaction buffer. Then, it was added 0.1124 μL of the recombinant enzyme in phosphate buffer and glycerol solution with concentration of 0.58 mg/mL (Bradford). Finally the reaction was started by the addition of 0.2 μmol of (+)-potassium D_s_-*threo*-isocitrate in solution.

Isoniazid was employed as a negative control (inhibition 0%), and 3-nitropropionic acid (3-NP) served as the positive control. All of the control compounds were added to the reaction mixture the same way like tested compounds and also their concentration in the reaction mixture was 10 μmol/L. The inhibitory activity of DMSO alone (1 μL) was subtracted from the activities of the evaluated compounds.

### 3.4. Antibacterial Evaluation

The *in vitro* antibacterial activity was assayed against eight Gram-positive and Gram-negative strains: *Staphylococcus aureus* CCM 4516/08, methicillin-resistant *Staphylococcus aureus* H 5996/08 (MRSA), *Staphylococcus epidermidis* H 6966/08, *Enterococcus* sp. J 14365/08; *Escherichia coli* CCM 4517, *Klebsiella pneumoniae* D 11750/08, ESBL-positive *Klebsiella pneumoniae* J 14368/08, and *Pseudomonas aeruginosa* CCM 1961.

The microdilution broth method modified according to standard M07-A07 in Mueller-Hinton broth (HiMedia Laboratories, Mumbai, India) adjusted to pH 7.4 (±0.2) was used. The tested compounds were dissolved in DMSO to the final concentrations ranging from 500 to 0.49 mmol/L. Benzylpenicillin (penicillin G) was used as a comparative drug. Bacterial inoculum in sterile water was prepared to match 0.5 McFarland scale (1.5 ± 10^8^ CFU/mL). The minimum inhibitory concentrations were assayed as 90% (IC_90_) or higher reduction of growth when compared to the control. The determination of results was performed visually and spectrophotometrically (at 540 nm). The values of MICs were determined after 24 and 48 h of incubation in the darkness at 35 °C (±0.1) in a humid atmosphere.

### 3.5. Cytotoxicity Evaluation (HepG2 Cells)

Seven esters and their parent salicylanilides with the most robust antimicrobial activity were tested for cytotoxicity in the human hepatocellular liver carcinoma cell line HepG2 (passages 21–24; ECACC, Salisbury, UK) using a standard colorimetric method measuring a tetrazolium salt reduction (CellTiter(R) 96 AQueous One Solution Assay, Promega G3580, Madison, WI, USA).

The cells were routinely cultured in Minimum Essentials Eagle Medium (Sigma-Aldrich, Darmstadt, Germany) supplemented with 10% foetal bovine serum (PAA; Biotech, Prague, Czech Republic), 1% of L-glutamine solution (Sigma-Aldrich), and non-essential amino acids solution (Sigma-Aldrich) in a humidified atmosphere containing 5% CO_2_ at 37 °C. For subculturing, the cells were harvested after trypsin/EDTA (Sigma-Aldrich) treatment at 37 °C. The cells treated with the tested substances were used as experimental groups. Untreated HepG2 cells were used as control groups.

The cells were seeded in density 1 × 10^4^ cells per well in a 96-well plate with microscopic control. Next day, the cells were treated with each of the tested substances. Because of the low solubility of some investigated compounds in water they were dissolved in a very small amount of DMSO and in a small volume added to cell culture; a final solution contained less than 1% DMSO. The most of the tested compounds were prepared in incubation concentrations of 0.01–20 µM in triplicates. 4-(Trifluoromethyl)benzoic acid was applied in the concentrations of 10–4,000 µM in triplicates. The following types of controls were included: determination of 100% viability and 0% viability (the cells treated by 10% DMSO), no cell control and vehiculum controls. These checking samples were prepared in triplicates.

The treated cells were incubated together with controls at 37 °C for 24 h in 5% CO_2_ atmosphere. After this time reagent from the kit CellTiter 96 AQueous One Solution Cell Proliferation Assay was added. The CellTiter 96 assay is based on the reduction of tetrazolium salt MTS [3-(4,5-dimethylthiazol-2-yl)-5-(3-carboxymethoxyphenyl)-2-(4-sulfophenyl)-2*H*-tetrazolium] to water-soluble formazan dye by metabolically active cells. The reduction of the reagent is attributed to availability of NADH or NADPH. The decline in levels of these metabolically important compounds in the cell causes that the production of formazan is reduced. The tested plate was incubated for 2 h at 37 °C and 5% CO_2_ and after this time, the absorbance was recorded at 490 nm using a 96-well plate reader (TECAN, Infinite M200, Grödig, Austria).

The results were expressed as inhibitory concentration which is necessary to inhibit cell viability to 50% from the maximal (control) viability (IC_50_). A standard toxicological parameter IC_50_ was calculated in each of the tested substances using GraphPad Prism software (version 5.02; GraphPad Software Inc., San Diego, CA, USA).

## 4. Conclusions

Eighteen salicylanilide 4-(trifluoromethyl)benzoates with a known antifungal activity underwent additional biological screening for the antimycobacterial and antibacterial activity, cytotoxicity and inhibition of mycobacterial isocitrate lyase. All compounds showed a significant antimycobacterial activity including against drug-resistant strains; in some cases comparable or even better than isoniazid. 3,4-Dichloro- and trifluoromethyl moieties represent the optimal substitution of aniline ring. Esters caused consistently a mild inhibition of isocitrate lyase; moreover, some derivatives are comparable to 3-nitropropionic acid. The substitution of aniline ring at the position 3 enhances this action. Although salicylanilide 4-(trifluoromethyl)benzoates failed with respect to our intention of finding the most antimycobacterial active salicylanilide esters, they offer very low MICs against Gram-positive strains and IC_50_ values for HepG2 cells.

In contrast to the aniline ring, the introduction of a 4-trifluoromethyl moiety into an acyl part of the salicylanilide esters did not provide sharply improved antimycobacterial activity, when compared to salicylanilide benzoates. The most potent esters are still considerably cytotoxic in micromolar values, although less than parent salicylanilides. Their cytotoxicity was mostly a slightly higher than for corresponding benzoates. In summary, salicylanilides and their 4-(trifluoromethyl)benzoates may be promising initial step compounds for anticancer research with advantageous broad-spectrum antimicrobial properties.
